# Case Report: Cervical chondrocalcinosis as a complication of Gitelman syndrome

**DOI:** 10.12688/f1000research.8732.1

**Published:** 2016-05-12

**Authors:** Zahra Iqbal, Paul Mead, John A. Sayer

**Affiliations:** 1 Renal Services, Newcastle upon Tyne Hospitals NHS Foundation Trust, Newcastle upon Tyne, UK; 2 Renal Unit, Cumberland Infirmary, Carlisle, UK; 3 Institute of Genetic Medicine, Newcastle University, Newcastle upon Tyne, UK

**Keywords:** chondrocalcinosis, Gitelman syndrome, hypomagnesaemia, musculoskeletal, SLC12A3

## Abstract

Gitelman syndrome is an inherited tubulopathy leading to a hypokalaemic metabolic alkalosis with hypomagnesaemia and hypocalciuria. Most cases are due to mutations in *SLC12A3*, encoding the apical thiazide sensitive co-transporter in the distal convoluted tubule. Musculoskeletal effects of Gitelman syndrome are common, including muscle weakness, tetany and cramps. Chronic hypomagnesaemia can lead to chondrocalcinosis, which often affects knees but can affect other joints. Here we present a case of Gitelman syndrome complicated by cervical chondrocalcinosis leading to neck pain and numbness of the fingers. Treatments directed at correcting both hypokalaemia and hypomagnesaemia were initiated and allowed conservative non-surgical management of the neck pain. Recognition of chondrocalcinosis is important and treatments must be individualised to correct the underlying hypomagnesaemia.

## Background

Gitelman syndrome (GS) is an autosomal recessive tubulopathy due to mutations in *SLC12A3* encoding the thiazide sensitive sodium chloride co-transporter (NCC) in the distal convoluted tubule. The estimated prevalence is 1:40,000 ^1^. Biochemically the phenotype is similar to long-term thiazide diuretic treatment: hypokalemia, hypomagnesemia, a hypochloraemic metabolic alkalosis and reduced urinary calcium levels ^2^. Although an inherited condition, the disease is usually diagnosed during adolescence or early adult life. However presentations late in life, often with chondrocalcinosis do occur ^3, 4^.

## Case report

A 55-year-old lady was referred to the renal unit with persistently low serum potassium and magnesium levels following an episode of acute cholecystitis. Urinary electrolytes confirmed potassium wasting and hypocalciuria. On admission, her serum electrolytes were deranged: potassium 2.5 mmol/L, magnesium 0.31 mmol/L, corrected calcium 2.04 mmol/L, sodium 134 mmol/L and creatinine 53 µmol/L. Additional biochemistry tests confirmed hyperreninaemic hyperaldosteronism (renin >14.4 pmol/ml/hr (NR 0.5–3.1) and aldosterone 2794 pmol/L (NR 100–800)). Random urine sodium was 97 mmol/l, urine potassium 33 mmol/l and urine osmolality 467 mosm/kg. Biochemically, the diagnosis was consistent with GS.

She previously had no other significant medical history but had required NSAIDs for longstanding back, hip and neck pain. She was commenced on oral potassium and magnesium supplements (magnesium oxide 16 mmol/day) together with spironolactone 100 mg daily as a long term treatment. Molecular genetic analysis confirmed GS with the identification of compound heterozygous mutations in *SLC12A3* (p.Arg209Gln and p.Ser615Leu) ^5^. Despite oral supplementation, serum magnesium levels remained low (0.5–0.6 mmol/L).

**Figure 1.**
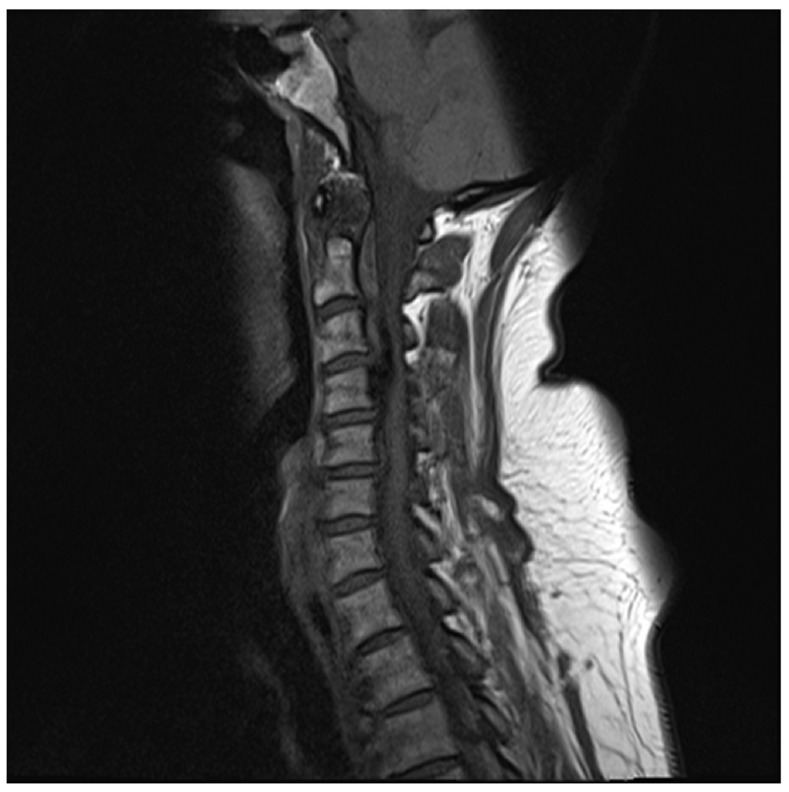
CT Cervical spine demonstrating a large ossified bony bar extending from the posterior surface of the C4 vertebral body up to the level of the upper surface of the C3 vertebral body. There are also multiple areas of calcification involving the intravertebral discs, annulus fibrosis, the ligmentum flavum and the transverse ligament behind the odontoid process.

At 60 years of age, she had a MRI spine examination for worsening neck pain and the onset of numbness in her fingers. The MRI spine revealed widespread chondrocalcinosis in the cervical spine and soft tissues, with a large ossified bony bar at the level of C3 and C4 compressing the spinal cord ( Figure 1). In addition, there were multiple areas of chondrocalcinosis in the intra-vertebral discs, annulus fibrosus, ligamentum flavum and in the transverse ligament behind the odontoid process. Despite the fingertip numbness and severe chondrocalcinosis, physical examination demonstrated no apparent neurological loss, with normal, tone, power, reflexes and sensation. Neurosurgical advice was sought and a conservative approach was adopted. Oral magnesium supplementation was changed to magnaspartate and increased to 40 mmol/day in an attempt to normalise serum magnesium levels and prevent progression of the chondrocalcinosis and improve symptoms. From 60 to 62 years of age the serum magnesium has been maintained at near normal levels (0.6–0.75 mmol/L) with improvement of musculoskeletal symptoms and no progression of any functional deficit in hand movements. Neurology follow-up continues to adopt observational and conservative management.

## Discussion

Chondrocalcinosis is the deposition of calcium pyrophosphate crystals in the articular cartilages throughout the body and has been associated with the longstanding hypomagnesaemia secondary to GS ^6^. Chondrocalcinosis may cause swelling, heat and tenderness over the affected joints. As well as GS, chondrocalcinosis may also be seen in association with hyperparathyroidism, haemochromatosis and hypophosphatasia.

Chondrocalcinosis is a known complication of GS and can affect various joints, most typically knees ^7^. Cervical spine chondrocalcinosis due to GS, however, is not often reported. Calcium pyrophosphate dehydrate deposits in the peri-odontoid soft tissues can lead to a condition called ‘crowned dens syndrome’ which causes acute neck pain and has been associated with GS ^8^. Treatment relies on magnesium replacement and symptom control with non-steroidal anti-inflammatory drugs. Surgery is rarely performed.

Patients with GS can experience salt craving, tetany and cramps, fatigue and severe lethargy, often impacting greatly on their quality of life ^5^. The chondrocalcinosis associated with it further adds to the musculoskeletal disease burden. It is therefore important to monitor patients and try to correct the potassium and magnesium disturbances to prevent acute exacerbations and progression ^9^. Complete normalisation of serum magnesium is often difficult due to diarrhoea associated with magnesium supplements. Various magnesium preparations are available, including magnesium oxide, magnesium glycerophosphate, magnaspartate, and magnesium lactate (Mag-Tab SR), which is slow release and is often better tolerated. Trial of various preparations and individual tailoring of dosing is required.

In summary we present a case of GS presenting with typical electrolyte disturbances (hypokalaemic metabolic alkalosis and hypomagnesaemia) complicated by severe chrondrocalcinosis of the cervical spine. Treatment directed at correcting these electrolyte disturbances has allowed an improvement of symptoms and avoidance of neurosurgery.

## Consent

Written informed consent was obtained from the patient for publication of this case report and any accompanying images and/or other details that could potentially reveal the patient’s identity.
