# Supplementary material for: Case Report: Cervical chondrocalcinosis as a complication of Gitelman syndrome
Source: F1000Res. 2016 May 12;5:875. [Version 1] doi: 10.12688/f1000research.8732.1 (PMC4897758; doi:10.12688/f1000research.8732.1)
Supplement: Peer review history

# Peer review: Referee response for version 1

**DOI:** 10.5256/f1000research.9396.r13933

**Published:** 2016-06-13

**Authors:** Andrew Mallett

**Affiliations:** 1 Department of Renal Medicine & Conjoint Statewide Renal Genetics Program, Royal Brisbane and Women's Hospital, Brisbane, Qld, Australia; 2 Australian Renal Gene Panels, Children's Hospital Westmead, Westmead, NSW, Australia; 3 School of Medicine, The University of Queensland, Brisbane, QLD, Australia; 4 Centre for Rare Diseases Research, Institute for Molecular Bioscience, The University of Queensland, Brisbane, QLD, Australia

This is a well constructed and executed Case Report which is informative both with regards to the uncommon complication and the primary inherited kidney disease it pertains to. It is likely to be of interest and utility for clinicians who may encounter a patient with Gitelman Syndrome. The background, case history and discussion are cohesive, add to the knowledge base and are important for advancing the understanding and clinical management of this rare disease.

I have read this submission. I believe that I have an appropriate level of expertise to confirm that it is of an acceptable scientific standard.

# Peer review: Referee response for version 1

**DOI:** 10.5256/f1000research.9396.r14146

**Published:** 2016-06-03

**Authors:** Nicholas G Larkins

**Affiliations:** 1 Princess Margaret Hospital, Perth, WA, Australia

A well written article describing an interesting presentation of an uncommon condition. The case would be useful to other clinicians, as there is some important discussion of magnesium preparations. 

 I note the authors reference another case report published this year by themselves of a 37 year female with chonedrocalcinosis and GS. These could have been combined, with a more substantial discussion for a more useful contribution to the literature.

I have read this submission. I believe that I have an appropriate level of expertise to confirm that it is of an acceptable scientific standard.

# Peer review: Referee response for version 1

**DOI:** 10.5256/f1000research.9396.r13809

**Published:** 2016-05-16

**Authors:** Coralie Bingham

**Affiliations:** 1 Renal Unit, Royal Devon and Exeter Hospital, Exeter, UK

A well written case report about an unusual complication of a rare disease. The genetic background to this disorder is covered. I think this report will be useful to other clinicians who may rarely see a case of Gitelman syndrome. There is a clear discussion of the medical management strategy used in this patient which lead to an improvement of symptoms and the avoidance of surgery. I think this report adds to knowledge about this rare disease and merits indexation.

I have read this submission. I believe that I have an appropriate level of expertise to confirm that it is of an acceptable scientific standard.
